# Cardiac Angiosarcoma from a Saphenous Vein Coronary Artery Bypass Graft

**DOI:** 10.7759/cureus.5503

**Published:** 2019-08-27

**Authors:** Pehr E Hartvigson, Gautam R Velamoor, Elizabeth T Loggers, Edward Kim

**Affiliations:** 1 Radiation Oncology, University of Washington Medical Center, Seattle, USA; 2 Cardiothoracic Surgery, Virginia Mason Medical Center, Seattle, USA; 3 Medical Oncology, University of Washington, Seattle, USA; 4 Radiation Oncology, University of Washington, Seattle, USA

**Keywords:** primary cardiac sarcoma, saphenous vein graft, autologous graft, cabg, coronary artery bypass graft, angiosarcoma

## Abstract

Primary cardiac sarcomas (PCS) are rare and the prognosis is generally poor. Angiosarcomas are the most common cardiac sarcoma histology. The best management of PCS is poorly defined. Chronic lymphedema, foreign bodies, including Dacron grafts, and arteriovenous fistulas have been associated with angiosarcoma, and angiosarcomas arising from saphenous vein femoropopliteal bypass grafts have been reported. We present the first known case of a cardiac angiosarcoma originating from an autologous saphenous vein graft used in a coronary artery bypass. The patient’s course and the literature on primary cardiac angiosarcomas are reviewed.

## Introduction

Primary cardiac sarcomas (PCS) are rare and account for about 0.85% of sarcomas [1]. The most common histology is angiosarcoma, comprising 32%-46% of all PCS [1-3]. PCS are characterized by aggressive clinical behavior. Optimal management of cardiac angiosarcomas is poorly defined given its rarity, but available evidence suggests the preferred primary treatment is surgery [1,3-6].

Although its pathogenesis remains debated, angiosarcoma has been associated with chronic lymphedema, arteriovenous fistulas, and Dacron grafts [7-12]. Several reports exist of angiosarcomas arising from saphenous vein autografts used in femoropopliteal bypasses [13-15]. Here, we present the first known case of an angiosarcoma originating from an autologous saphenous vein graft used in a coronary artery bypass graft (CABG).

## Case presentation

**Figure 1:**
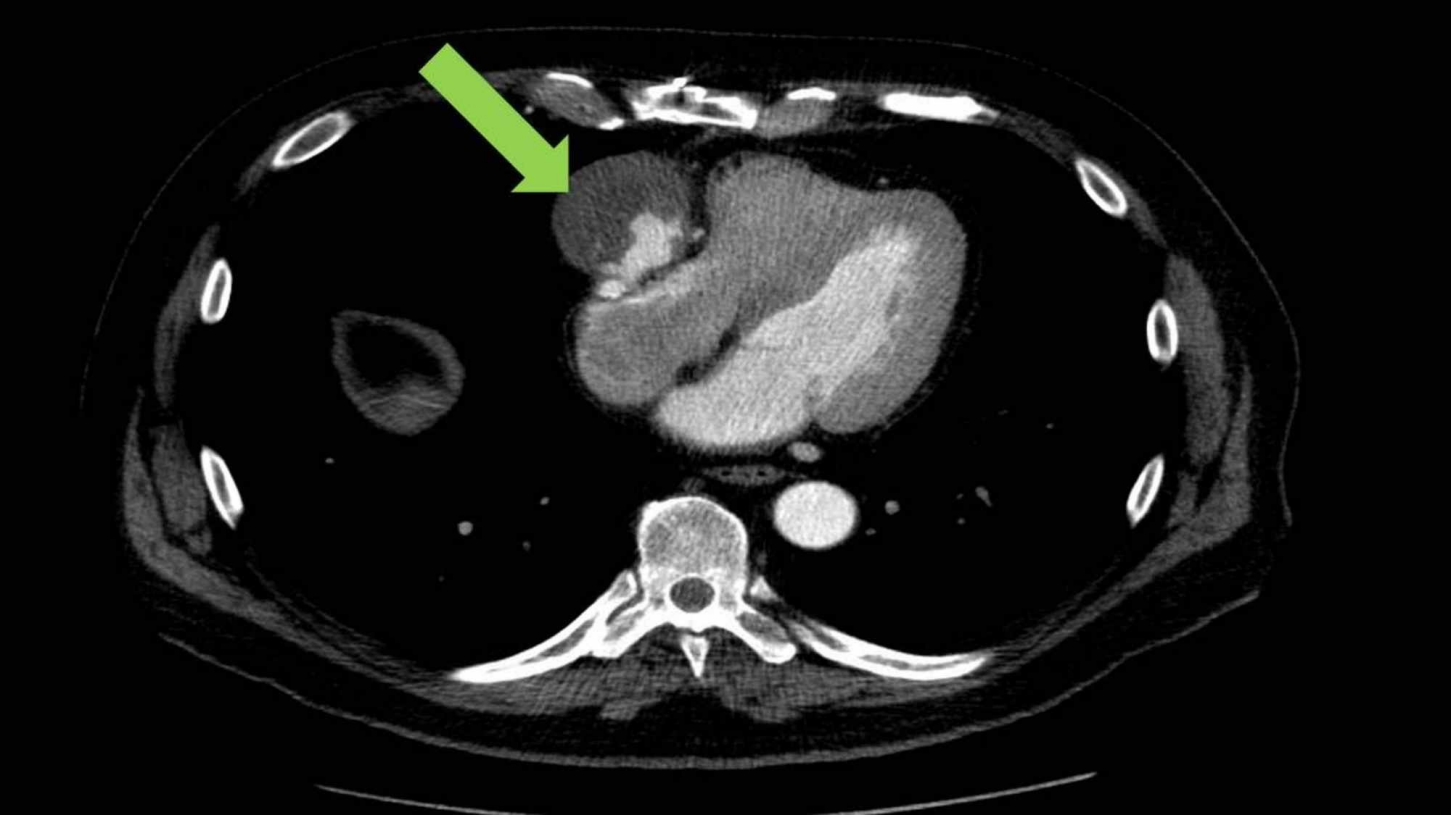
CT angiogram at diagnosis Green arrow indicates a pseudoaneurysm

The patient was a 72-year-old man with a history of coronary artery disease treated with four-vessel CABG in 1996. In September 2014, he presented to an outside emergency department with acute onset of substernal chest pain. New-onset atrial fibrillation was diagnosed. His troponin and chest X-ray were normal. Electro-cardioversion performed in the emergency department restored normal sinus rhythm. Coronary angiography revealed severe and moderate stenosis of four native arteries and an ectatic and unusual-appearing saphenous graft to the posterior descending artery. The differential diagnosis included aneurysm, pseudoaneurysm, and arteriovenous malformation. Computed tomography (CT) angiogram demonstrated the saphenous graft extending from the aorta to the right coronary artery with a 5 x 6 x 6 cm pseudoaneurysm near the coronary anastomosis with a large associated thrombus and extrinsic compression of the right atrium (Figure 1).

**Figure 2:**
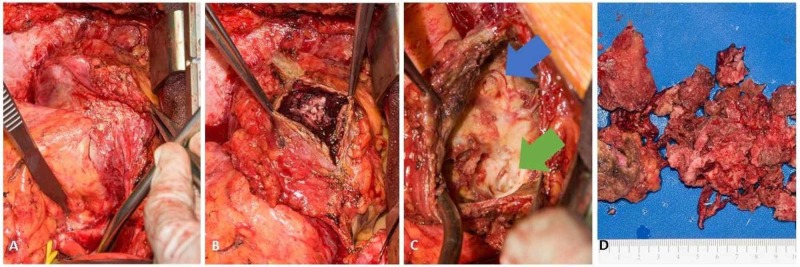
Intra-operative findings and gross specimen A) Pseudoaneurysm with pericardial adhesions, B) Dissection revealing thrombus, C) Dissected pseudoaneurysm; green arrow indicates the saphenous graft aortotomy site and the blue arrow indicates the posterior descending artery anastomosis site, D) Evacuated thrombus

Repeat sternotomy, two-vessel CABG, and clot evacuation were performed. Intraoperatively, the pseudoaneurysm measured 10 cm and was adjacent to the right atrium with substantial pericardial adhesions (Figure 2A). The clot was evacuated (Figures 2B-2D). The pseudoaneurysm remained in situ and a new saphenous graft was laid in the splayed pseudoaneurysm bed. The patient tolerated the operation well and his postop course was unremarkable. Pathology found irregular clusters of malignant cells lining the surface of the thrombus, which were immunostain-positive for AE1/3, CD-31, and CD-34, consistent with an epithelioid angiosarcoma. An outside academic pathology department with expertise in sarcoma reviewed the case and confirmed the diagnosis. There was no evidence of regional or distant metastases on PET-CT. The resection bed demonstrated residual soft tissue and complex fluid density in the area of the repaired pseudoaneurysm with moderate fluorodeoxyglucose (FDG) avidity, which were attributed to post-surgical changes and wound healing. No gross residual disease was present. The patient was referred to our institution for specialized oncology care. The pathology was reviewed by a sarcoma-specialized pathologist at our hospital with concordant findings.

**Figure 3:**
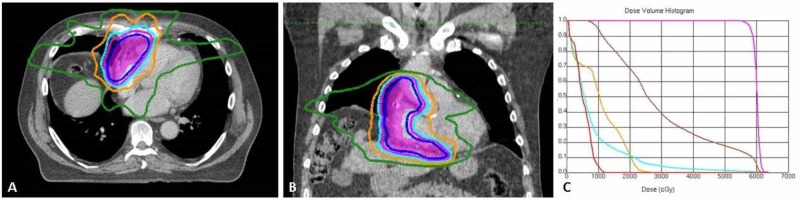
Radiotherapy plan and dose volume histogram A) Axial view, B) Coronal view, C) Dose volume histogram. In A and B: Magenta-shaded region = planning target volume (PTV); solid blue line = 95% isodose line (57 Gy); Magenta line = 50.4 Gy; Sky-blue line = 45 Gy; Orange = 35 Gy; Green = 20 Gy. In C: Magenta = PTV; Brown = Heart; Orange = Esophagus; Sky-blue = Lung, total; Red = Spinal canal

The multidisciplinary tumor board recommended adjuvant radiotherapy without chemotherapy. Four-dimensional CT was used for treatment planning to account for organ motion, and a plan was designed to deliver 60 Gy in 30 fractions using seven-field static beam intensity-modulated radiotherapy (Figures 3A-3B). This radiation dose was selected given the extent of heart tissue (right atrium and ventricle) that was incorporated in the radiation target volumes. The clinical target volume (CTV) was derived from a discussion with the cardiac surgeon and a review of the operative report, photos of the surgical specimen, post-operative PET-CT, and post-operative CT-cardiac angiogram. The CT angiogram was fused to the treatment planning CT to identify the pathway of the involved bypass graft (starting at the insertion to the ascending aorta), and a 1.5 cm radial margin expansion was added, not extending into the lung, bowel, liver, or uninvolved chambers of the heart. The entire operative bed, including the involved retrosternal fat and pericardium, was included in the CTV. The final CTV abutted the lateral walls of the right ventricle and right atrium. The planning target volume (PTV) consisted of a 5 mm universal expansion of the CTV. The dose-volume objectives for targets and organs at risk were met (Figure 3C and Table 1). The patient tolerated therapy well and continued his cardiac rehabilitation program throughout radiotherapy. Post-treatment surveillance consisted of a CT chest every three months.

**Table 1:** Organ at risk dose statistics V60 Gy = percent organ volume at 60 Gy, etc.; D50% = dose to 50% of the organ

Organ	Goal	Treatment Plan
Heart	V60 Gy < 30%	V60 Gy = 6%
	V45 Gy < 60%	V45 Gy = 21%
Left ventricle	D50% < 35 Gy	D50% = 18.6 Gy
Esophagus	Mean < 35 Gy	Mean = 9.9 Gy
Lung, total	V20 Gy < 20%	V20 Gy = 11%
	V10 Gy < 40%	V10 Gy = 24%
	V5 Gy < 60%	V5 Gy = 52%
Spinal canal	Max dose < 45 Gy	Max dose = 12 Gy
Large bowel	Max dose < 50.4 Gy	Max dose = 50.1 Gy

In April 2016, he presented with hemoptysis and a CT chest revealed a new 4.5 cm left hilar mass, several suspicious mediastinal lymph nodes, and multiple enlarged bilateral pulmonary nodules. The primary site was stable. Bronchoscopy demonstrated an obstructive mass in the lingular bronchus; biopsy was positive for angiosarcoma. He tolerated a palliative radiotherapy course directed at the new mass of 40 Gy in 10 fractions well. His hemoptysis resolved by treatment completion. However, cancer rapidly progressed, and he presented several weeks later with recurrent hemoptysis attributed to two, new, large, right peripheral lung masses, which were treated with a single fraction of 8 Gy. He desired to attempt systemic therapy, and paclitaxel was recommended. He received only one infusion in June 2016 before he was admitted 1.5 weeks later for hemoptysis and an ST-elevated myocardial infarction. His hospital course was complicated by sepsis. Ultimately, he selected comfort-focused care and died at home surrounded by family several weeks later, at 21 months from his initial resection.

## Discussion

Primary cardiac tumors are extremely rare, with an estimated incidence of 0.02%. About a quarter of all tumors are malignant [16]. Sarcomas are the most common primary cardiac malignancy, with angiosarcoma accounting for 35%-39% of PCS [2,17-18]. Ab cardiac angiosarcoma is characterized by aggressive clinical behavior: median overall survival between six and 17.5 months has been reported [1,18], and four-year overall survival in one multi-institutional series was 8.4% for angiosarcomas vs. 28.8% for other PCS histologies [3].

Angiosarcomas have been known to arise from chronic lymphedema [10], Dacron vascular grafts [7,9,11], and arteriovenous fistulas [8,12]. The pathogenesis of angiosarcoma is unknown, but speculated etiologies include regional immunosuppression from chronic lymphovascular congestion in Stewart-Treves Syndrome, chronic inflammation to foreign bodies, and changes in the vascular wall shear-stress in fistulas. Three instances of angiosarcoma from venous grafts in femoropopliteal bypasses have been described [13-15]. The etiology of saphenous graft malignant transformation is thought to be a combination of angiolymphatic congestion and local hemodynamic changes. Means to mitigate the risk of malignant transformation remain unknown.

The optimal management of PCS is not well-defined due to its rarity, and clinical data is limited to case series. These series indicate the central role of complete resection. Median survival for patients who had negative (R0) or microscopically positive margins (R1) was 23.5 months in one surgical series [4]. Notably, 30% of the patients required cardiac explantation and autotransplantation to achieve R0/R1 margins, and two patients died perioperatively. A study of the Surveillance, Epidemiology, and End Results (SEER) database showed a 12-month median survival for patients treated with surgery vs 1 month without surgery [1]. Median survival was 38.8 months for patients with R0 vs 18.2 months with R1 or macroscopic (R2) incomplete resection in a French Sarcoma Group analysis of 124 patients [3]. Similarly, Wu et al. found that R0/R1 was significantly associated with improved overall survival (21.8 vs 7.2 months if R2) [6]. Surgical resection was statistically significant on univariate, but not multivariate, analysis of 62 patients from a multinational case series [18]. Highlighting the surgical complexity of these cases, only 8% of patients had R0 resections in this international cohort. Several cases of definitive radiotherapy for unresectable tumors have been described with durable local control. Techniques have included hyperfractionation with a radiosensitizer [19] and stereotactic body radiotherapy (SBRT) with concurrent paclitaxel [20].

The role of adjuvant therapy is less well-defined. Adjuvant therapy (chemotherapy or radiotherapy) was significantly associated with improved survival in an early study [2]. The SEER study showed a non-significantly improved median survival of 11 months vs four months with vs. without radiation [1]. In the French Sarcoma Group study, 15% of patients received adjuvant radiotherapy, and radiotherapy significantly improved progression-free survival on the multivariate analysis but not overall survival [3]. The median cumulative radiation dose in this study was 50 Gy. Chemotherapy significantly improved overall survival in metastatic but not non-metastatic patients in the French Sarcoma Group study [3]. The Stanford case series found a non-significant improvement in overall survival with postoperative therapy of 15.5 vs. 2.6 months [6]. Patients received various combinations of chemotherapy (primarily either doxorubicin/ifosfamide or gemcitabine/paclitaxel combinations) and radiation (dose range 43.5-55.8 Gy). Multi-modality therapy did not significantly impact prognosis in the multinational series [18]. In the present case, 60 Gy of adjuvant radiotherapy was given in standard fractionation, and chemotherapy was deferred to the time of progression, in part because of medical comorbidities and lack of known overall survival benefit in the adjuvant setting. Ultimately, paclitaxel was chosen.

## Conclusions

To the best of our knowledge, this is the first account of a cardiac angiosarcoma arising from a saphenous vein autograft used for CABG. This report adds to three known cases of angiosarcoma arising from saphenous vein grafts bypassing non-coronary vessels. Oncologic resection was not performed, as there was no suspicion of malignancy upfront. Nevertheless, partial resection and adjuvant radiotherapy provided clinical local control. Across multiple case series, R0/R1 resection impacts survival, but the ideal adjuvant therapy regimen remains elusive, and most patients (including the patient presented here) succumb to distant metastases. Further collaboration is needed to establish the ideal therapeutic regimen and to improve the generally dismal prognosis of cardiac angiosarcoma.
